# Maternal determinants of low birth weight among Indian children: Evidence from the National Family Health Survey-4, 2015-16

**DOI:** 10.1371/journal.pone.0244562

**Published:** 2020-12-31

**Authors:** Ankita Zaveri, Pintu Paul, Jay Saha, Bikash Barman, Pradip Chouhan

**Affiliations:** 1 Department of Geography, University of Gour Banga, Malda, West Bengal, India; 2 Centre for the Study of Regional Development, School of Social Sciences, Jawaharlal Nehru University, New Delhi, India; Institute of Economic Growth, INDIA

## Abstract

**Objective:**

Low birth weight (LBW) is a serious public health problem in low- and middle-income countries and a leading cause of death in the first month of life. In India, about 18% of children are born with LBW (<2500 grams) in 2015–16. In this study, we aim to examine the influence of maternal factors and socio-demographic covariates on LBW in Indian children.

**Methods:**

Data were drawn from the fourth round of the National Family Health Survey (NFHS-4), conducted in 2015–16. A cross-sectional study was designed using a stratified two-stage sampling technique. Cross-tabulation, Pearson’s chi-squared test, and multivariate logistic regression analyses were employed to assess the impact of maternal factors and other covariates on children’s LBW.

**Results:**

Of total participants (n = 147,762), 17.5% of children were found to be born with LBW. The study revealed that women who had prior experience of stillbirth (Adjusted odds ratio [AOR]: 1.20, 95% CI: 1.04–1.38) and any sign of pregnancy complications (AOR: 1.08, 95% CI: 1.05–1.11) were more likely to have LBW children, even after adjusting for a range of covariates. Maternal food diversity was found to a protective factor against children’s LBW. Women with underweight and anemic condition were associated with an increased likelihood of LBW children. Regarding maternity care, women who attended ≥4 ANC visits (AOR: 0.84, 95% CI: 0.80–0.88), took iron tablets/syrup during pregnancy (AOR: 0.94, 95% CI: 0.90–0.98), and delivered in a public health facility (AOR: 0.84, 95% CI: 0.79–0.88) were less likely to have LBW babies. Besides, various socio-demographic factors such as place of residence, caste, religion, education, wealth quintile, and geographical region were significantly associated with LBW of children.

**Conclusion:**

Interventions are needed for adequate ANC utilization, improvement in public facility-based delivery, providing iron supplementation, and uptake of balanced energy-protein diet among pregnant mothers. Besides, special attention should be given to the socio-economically disadvantaged women to address adverse pregnancy and birth outcomes including LBW.

## Introduction

The first weight of newborns, usually taken just after birth is called birth weight. The World Health Organization (WHO) defines low birth weight (LBW) as weight at birth below 2500 grams or 5.5 pounds regardless of gestational age [1]. LBW can be categorized as very low birth weight (<1500 grams) and extremely low birth weight (<1000 grams) [1]. LBW is a major public health problem and a leading risk factor of childhood morbidity and mortality. It is reported that 60–80% of neonatal deaths have been occurred due to LBW worldwide. Every year, about 20.5 million newborns are born with LBW, accounting for 14.6% of total infants in the world. Among them, 96.5% of LBW infants are born in developing countries [1,2].

In 2015, the incidence of LBW is found to be highest in South Asia (27%) than any other region in the world. The prevalence of LBW is almost stagnant in all regions of the world between 2000 and 2015 [2]. India has witnessed a high burden of childhood malnutrition and mortality [3,4]. Although India has made considerable progress in reducing LBW of children during the past decade, it remains a leading cause of child mortality in the country, especially among socio-economically disadvantaged groups [5]. According to the latest National Family Health Survey (NFHS-4), about 18% of Indian children younger than five years are born with LBW in 2015–16 [6]. To achieve the target of Sustainable Development Goal (SDG) in infant and under-5 mortality levels by 2030, an accelerated improvement is still required in reducing the occurrence of LBW.

Infants with LBW are more likely to suffer from stunted growth. It affects children’s schooling outcomes, productivity, and cognitive development. It is also associated with increased risks of infections, childhood illnesses, long-term physical and mental disorders, and other chronic diseases in later part of life [7,8].

Previous research has documented that LBW of children is determined by a complex interplay of biological, socioeconomic, obstetric, maternity care during pregnancy, nutrition, and environmental factors [9–13]. For instance, a recent study conducted in India using two consecutive large-scale sample surveys found a significant influence of biological characteristics, mothers’ nutrition, programmatic factors, and socio-economic status on the occurrence of LBW [10]. Their study has revealed that mothers’ daily consumption of nutritional foods, adequate utilization of antenatal care (ANC) and delivery care, and improved socio-economic status were protective against LBW of children [10]. Of particular interest, an earlier study of India indicates that the nutritional status of the mother, measured from body mass index (BMI), is the most important determinant of LBW in children [11]. Similarly, a study carried out in Bangladesh also reported the importance of mothers’ schooling and adequate use of ANC and delivery care in reducing the risk of LBW babies [12]. Proper care during pregnancy is critical for healthy growth and development of the fetus by providing essential services, clinical examination, and counseling to the mothers regarding several aspects of pregnancy including consumption of nutritional foods. A recent study of India found a strong association between ANC of mothers and the birth weight of children [13]. Furthermore, a multi-country study revealed that mothers’ short height, illiteracy, anemia, inadequate ANC visits, and poor nutritional status were associated with an elevated risk of LBW babies [8].

**Fig 1:**
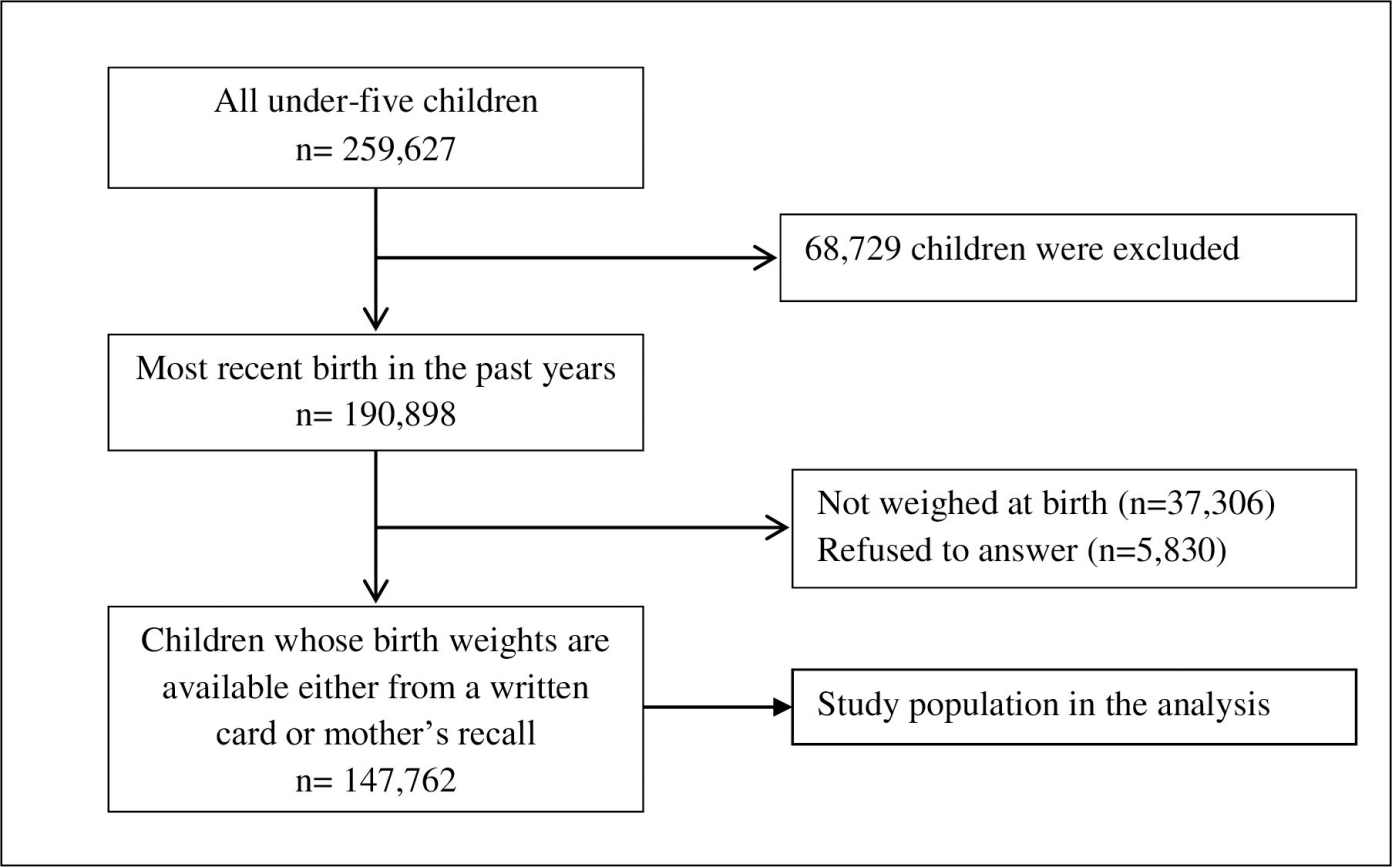
Selection of study participants, NFHS-4.

It is understood from prior evidence that the occurrence of LBW is largely determined by reproductive behaviors, food consumption, nutritional status, provisioning of maternity care during pregnancy, access to healthcare services, and socio-economic status of the mother. Among all the factors identified from past research, maternal factors are found to be the most important determinants of LBW. We conceptualize that LBW of children is influenced by a set of key maternal factors. Moreover, there are several underlying background factors (e.g., education, caste, religion, wealth quintile, etc.) that would determine the LBW of children through these proximate maternal factors (Fig 1).

Although there is a plethora of research on various factors of LBW in children, less attention has given to the factors related to maternal characteristics, especially in Indian context. Our study contributes to significant evidence in the growing body of research on proximate maternal determinants of LBW in children that would be useful to design policies and programs to combat the high incidence of LBW children. Therefore, we aim to investigate the influence of key maternal factors and underlying socio-demographic factors on LBW among Indian children, using a large-scale nationally representative cross-sectional sample survey.

## Materials and methods

### Data source

Data were drawn from the fourth round of the National Family Health Survey (NFHS-4), conducted in 2015–16. The NFHS-4 is a large-scale nationally representative sample survey, carried out across all Indian states and union territories (UTs) under the stewardship of the Ministry of Health and Family Welfare (MoHFW), Government of India, and the International Institute for Population Sciences (IIPS), Mumbai. The data collection was completed for 601,509 households, 699,686 women aged 15–49 years with a response rate of 97%, and 112,122 men aged 15–54 years with a response rate of 92%. The primary objective of this survey was to provide updated and reliable information on fertility, family planning, maternal and child healthcare, childhood immunization, infant and child mortality, nutrition, HIV/AIDS-related knowledge and attitudes, women empowerment, and domestic violence against women. A stratified two-stage sampling technique was adopted for the collection of data. In the first stage, clusters were selected using probability proportional to the cluster size. In the second stage, 22 households from each cluster were selected with an equal opportunity systematic selection from the household listing. A detailed description of the sampling design and survey procedure is provided in the national report of NFHS-4 [6].

### Study participants

**Fig 2:**
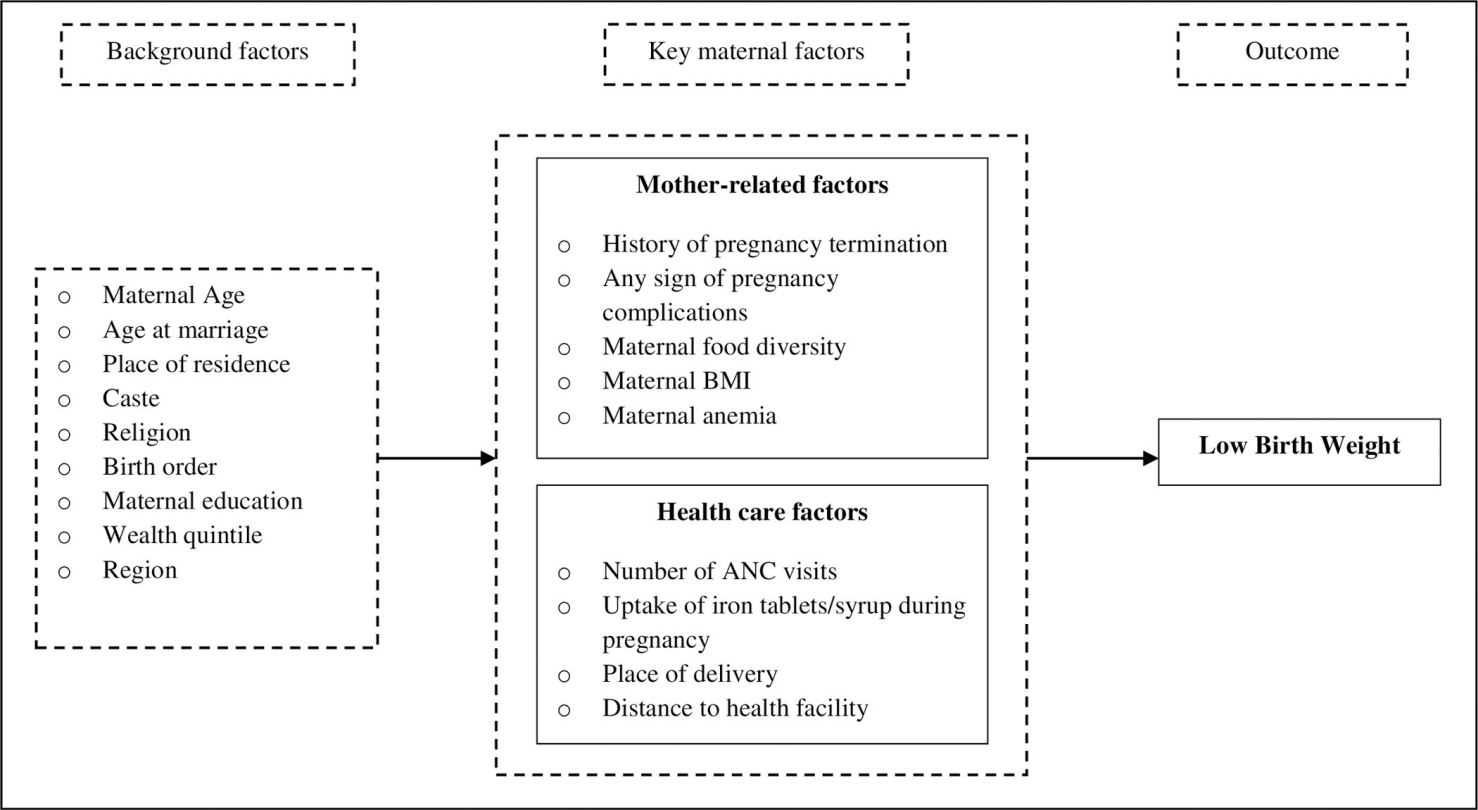
Conceptual framework showing maternal determinants of low birth weight.

The NFHS-4 provides information about 259,627 children born to women aged 15–49 years in the past five years preceding the survey. To avoid recall bias, this study was limited to the most recent births in the past five years (n = 190,898). Among them, 37,306 children were not weighed at birth and 5830 respondents were refused to answer about children’s birth weight. Therefore, the final analytical sample was limited to 147,762 most recent births in the past five years preceding the survey (Fig 2).

### Outcome variable

The low birth weight of children is considered as the outcome variable in this study. The birth weight of children was categorized as <2500 grams (low birth weight) and ≥2500 grams (normal birth weight). The information on the birth weight was collected either from mothers’ recall or a written record of health cards. Children were coded as ‘1’ if they had less than 2500 grams of the birth weight; otherwise, they were coded as ‘0’.

### Key explanatory variables

In this study, we aim to examine the impact of key maternal factors on LBW in children. Furthermore, we controlled a wide range of socio-demographic factors as to understand how covariates alter the influence of maternal factors in determining LBW in children. The selection of these variables is based on past research on LBW conducted in India and other developing countries. The key maternal factors are grouped into two broad categories: (a) mother-related factors and (b) health care factors.

Concerning mother-related factors, we considered reproductive health, food consumption, and nutritional status of the mother in the analysis. It is understood from the previous literature that these maternal factors are prominent determinants of children’s LBW. Mother-related factors include history of pregnancy termination (categorized as miscarriage, abortion, stillbirth, and none), any sign of pregnancy complications (no/yes), maternal food diversity index (low/medium/high), maternal BMI (underweight/normal/overweight or obese), and maternal anemia (not anemic/anemic).

Food diversity index was measured from the eating frequency of nine food items that were asked to the mothers: milk or curd, pulses or bean, dark green leafy vegetables, fruits, eggs, fish, chicken or meat, fried food, and aerated drinks. The survey responses were recorded as daily, weekly, occasionally, and never for each food item. The distribution of study participants by the consumption of food items can be found in S1 Table. All the variables related to food are dichotomized as the following two groups: (a) respondents who were having daily or weekly considered as consumers (coded as ‘1’), and (b) eating occasionally and never were grouped as non-consumers (coded ‘0’). We measured food diversity scores by combining all food items and grouped into three levels of food diversity index: low (consumed <3 food items), medium (consumed 3–6 food items), and high (consumed 7–9 food items). BMI is a measure of nutritional status among adults. The NFHS-4 provided anthropometric data related to the height and weight of women. Maternal BMI was computed by weight in kilograms divided by height in meters squared (kg/m^2^). We used WHO cut-off points to classify the BMI of mothers as three groups: <18.5 kg/m^2^ (underweight), 18.5–24.9 kg/m^2^ (normal) and ≥25.0 kg/m^2^ (overweight/obese). Anemia is a measure of the amount of hemoglobin present in the blood or red cell count in the blood. If women recorded hemoglobin level below 11.0 grams/decilitre (<12.0 grams/decilitre for pregnant women) were considered as anemic. Hemoglobin levels also adjusted for cigarette smoking and altitude in enumeration areas that were above 1,000 meters.

In regard to health care factors, our study included both the demand-side and supply-side factors of maternal health care. These factors are the number of ANC visits (none/1–3/≥4), uptake of iron tablets/syrup during pregnancy (no/yes), place of delivery (home/public facility/private facility), and distance to health facility (no problem/big problem/not a big problem).

### Covariates

Demographic and socio-economic factors were considered as covariates in this study. The selection of these factors is based on previous studies conducted on LBW in India and other countries. Demographic factors include women’s age (15–24, 25–34, and 35–49 years) and age at marriage (<18 and ≥18 years). Furthermore, socioeconomic factors include the place of residence (urban and rural), caste (forward caste, scheduled caste, scheduled tribe, and other backward classes), religion (Hindu, Muslim, and others), birth order (one, two, and three or more), maternal education (no education, primary, secondary, and higher), wealth quintile (poorest, poorer, middle, richer, and richest), and region (north, central, east, northeast, west, and south).

### Statistical analyses

Univariate statistics, bivariate, and multivariate logistic regression were carried out for the analyses of data in this study. Descriptive statistics were performed to show the distribution of study participants. Bivariate analyses were carried out to understand the nature of the association between predictors and the outcome variable. The test of association was performed using Pearson’s chi-squared statistic. The sample weight was used to estimate the percentages. Furthermore, a series of three binary logistic regression models were employed to assess maternal factors associated with children’s LBW. The first model assessed the influence of mother-related factors on LBW of children. In the second model, health care factors were progressively included. Finally, demographic and socio-economic factors were incorporated into a single model to determine the impact of maternal factors on children’s LBW independent of other covariates. We checked for multicollinearity between independent variables before performing the multivariate logistic regression. In this study, multicollinearity was tested at two stages. In the first stage, a correlation matrix was developed based on nine food items, which have been used for the construction of food diversity index, to examine whether these food items were highly correlated with each other or not (S2 Table). In the second stage, collinearity was tested using variance inflation factor (VIF) technique between all the selected explanatory variables (S3 Table). We found no collinearity in these variables. The regression results are presented by estimated odds ratios (ORs) and 95% confidence intervals (CIs). The level of significance was shown by *p* values and it was set at *p*<0.05. All the statistical analyses were performed using STATA version 12.1 (StataCorp LP, College Station, TX, USA).

### Ethical statement

The ethical approval of the National Family Health Survey (NFHS-4) was obtained from the ethics review board of the International Institute for Population Sciences (IIPS), Mumbai, India. This survey was also reviewed and approved by ICF International Review Board (IRB). Informed written consent for participation in this survey was obtained from the respondents during the survey. Each individual's approval was sought, and then only the interview was conducted. The NFHS-4 is an anonymous publicly available dataset with no identifiable information of the survey participants and accessible upon a granted request from the Demographic and Health Surveys (DHS) Program at https://dhsprogram.com/data/available-datasets.cfm.

## Results

### Participant’s characteristics

Of the total respondents (n = 147,762) about 17.5% of children were born with LBW. The majority of women had no history of pregnancy termination (88.8%) and no signs of pregnancy complications (58%). About two-thirds of respondents (63.8%) had a medium level of diversity in their meals. Nearly one in every four women (22.7%) was underweight and over half of them (55.6%) were anemic. About 60% of women had received ≥4 ANC visits. An overwhelming majority of women took iron tablets or syrup during pregnancy (84.7%) and delivered in either public or private health facilities (93.1%). A considerable proportion of women reported either no problem (36.3%) or not a big problem (34.7%) regarding distance to the health facility. Regarding socio-demographic characteristics, over half of the women (55.9%) belonged to age 25–34, and more than one in every three women (36.2%) married before they reached 18 years. Most of the respondents lived in rural areas (66.8%), belonged to other backward classes (45.1%), and affiliated to Hindu religion (80.2%). Over half of the women (52.2%) had a secondary level of education. Participants were almost equally distributed across household wealth quintiles. The highest proportion of respondents resided in the east region (23.7%), followed by the south (22.6%), and central (20.9%) regions of the country (Table 1).

**Table 1:** Respondent’s characteristics and birth weight of children by explanatory variables, NFHS-4 (n = 147,762).

Variables	Frequency (%)	Birth weight (%)		*p*-value
		<2.5 kg (low birth weight)	≥2.5 kg (normal birth weight)	
**Mother-related factors**				
Pregnancy termination				<0.001
Miscarriage	9877 (6.4)	18.1	81.9	
Abortion	4206 (3.0)	17.8	82.6	
Stillbirth	1302 (0.8)	20.4	79.6	
None	132,377 (89.8)	17.4	82.6	
Any sign of pregnancy complications				<0.001
No	84,488 (58.0)	17.3	82.8	
Yes	63,020 (42.0)	17.8	82.2	
Food diversity index				<0.001
Low	24,799 (14.1)	20.4	79.6	
Medium	95,233 (63.8)	17.5	82.6	
High	27,730 (22.1)	15.8	84.2	
Maternal BMI				<0.001
Underweight	32,130 (22.7)	21.3	78.7	
Normal	89,400 (59.5)	16.8	83.2	
Overweight/obese	24,203 (17.7)	14.6	85.4	
Maternal anemia				<0.001
Not anemic	66,079 (44.4)	17.1	82.9	
Anemic	79,136 (55.6)	17.8	82.2	
**Health care factors**				
Number of ANC visits				<0.001
None	16,344 (10.9)	20.3	79.7	
1–3	48,606 (29.6)	18.3	81.7	
≥4	81,292 (59.5)	16.6	83.4	
Uptake of iron tablets/syrup during pregnancy				<0.001
No	24,456 (15.3)	20.0	80.0	
Yes	122,913 (84.7)	17.0	83.0	
Place of delivery				<0.001
Home	11,173 (6.8)	19.8	80.2	
Public health facility	96,812 (60.0)	17.6	82.4	
Private health facility	39,480 (33.2)	16.8	83.2	
Distance to health facility				<0.001
No problem	52,109 (36.3)	16.4	83.6	
Big problem	44,227 (29.0)	19.0	81.0	
Not a big problem	51,426 (34.7)	17.4	82.6	
**Covariates**				
Women's age (years)				<0.001
15–24	50,479 (36.4)	19.0	81.0	
25–34	83,511 (55.9)	16.5	83.5	
35–49	13,772 (7.7)	17.3	82.7	
Age at marriage (years)				<0.001
<18	49,449 (36.2)	18.6	81.4	
≥18	95,868 (63.8)	16.9	83.1	
Place of residence				0.001
Urban	41,909 (33.2)	16.7	83.3	
Rural	105,853 (66.8)	17.9	82.1	
Caste				
Forward caste	28,879 (22.9)	16.2	83.8	<0.001
Scheduled caste	27,071 (21.7)	18.4	81.6	
Scheduled tribe	26,626 (10.3)	19.5	80.5	
Other backward classes	58,309 (45.1)	17.1	82.9	
Religion				<0.001
Hindu	110,994 (80.2)	17.8	82.2	
Muslim	19,979 (14.4)	16.6	83.4	
Others	16,789 (5.4)	15.9	84.2	
Birth order				<0.001
1	53,876 (37.3)	18.2	81.8	
2	51,297 (36.5)	16.6	83.4	
≥3	42,589 (26.2)	17.7	82.3	
Maternal education				<0.001
No education	32,581 (20.8)	19.6	80.5	
Primary	19,485 (12.8)	19.7	80.3	
Secondary	76,610 (52.2)	17.3	82.7	
Higher	19,086 (14.2)	13.3	86.7	
Wealth quintile				<0.001
Poorest	27,844 (17.5)	19.6	80.4	
Poorer	31,579 (20.2)	18.4	81.6	
Middle	31,619 (21.2)	17.8	82.2	
Richer	29,594 (21.5)	17.7	82.3	
Richest	29,594 (19.6)	14.1	85.9	
Region				<0.001
North	29,149 (13.7)	20.0	80.1	
Central	36,336 (20.9)	19.5	80.5	
East	29,529 (23.7)	15.7	84.3	
Northeast	20,398 (3.7)	13.9	86.0	
West	13,011 (15.5)	18.5	81.6	
South	19,339 (22.6)	16.0	84.0	
**Overall**	**147,762 (100.0)**	**17.5**	**82.5**	

### Prevalence of LBW by explanatory variables

The occurrence of LBW was found to be higher among women who had a history of pregnancy termination (miscarriage: 18.1%; abortion: 17.8; stillbirth: 20.4%) and any sign of pregnancy complications (17.8%) as compared to their counterparts. Mothers who had low diversity in diet considerably had a higher incidence of LBW babies (20.4%) than those mothers who had medium (17.5%) or high (15.8%) diversity in their meals. Furthermore, women experiencing underweight (21.3%) and anemia (17.8%) were more likely to have LBW children compared to their counterparts. The incidence of LBW babies was lower among women who attended ≥4 ANC visits (16.6%), took iron tablets or syrup during pregnancy (17%), and delivered in a health institution (public: 17.6%; private: 16.8%). Respondents who faced difficulties regarding distance to the health facility had a higher prevalence of LBW children (Table 1).

The prevalence of LBW decreased with the increase of the age of women in which women aged 15–24 years had a higher proportion of LBW babies than those women aged 35 years or older (19% vs. 17.3%). A higher percentage of mothers had LBW children who married before 18 years compared with those who married at 18 years or later (18.6% vs. 16.9%). The incidence of LBW decreased with the increase of maternal educational level. The prevalence of LBW was 6.3% lower among higher educated women as compared to women who had no formal education (13.3% vs. 19.6%). The occurrence of LBW also had a decreasing trend from bottom to upper quintiles of household wealth where the percentage of LBW was 5.5% lower in the richest quintile than the poorest household. Children’s LBW was common among those who lived in rural areas (17.9%), belonged to scheduled caste (18.4%) and scheduled tribe (19.5%) and Hindus (17.8%). The prevalence of LBW also varied across geographical regions. The highest incidence of LBW was found in the north region (20%), followed by the central (19.5%) and west (18.5%) regions (Table 1).

### Maternal determinants of LBW in children

The results of binary logistic regression for assessing the impact of maternal factors on LBW in children are presented in Table 2. In the first model, we employed mother-related factors such as pregnancy termination, pregnancy complications, maternal food diversity, maternal BMI, and maternal anemia that were significantly associated with children’s LBW. However, the odds of these factors were slightly modified when we included health care factors in the second model. For instance, while the likelihood of pregnancy termination marginally increased, the influence of food diversity index and maternal BMI reduced in the second model. Moreover, maternal factors remain pertinent even after adjusting for socio-demographic covariates in the final model. Final model indicates that women who had prior experience of stillbirth (Adjusted odds ratio [AOR]: 1.20, 95% CI: 1.04–1.38) and any sign of pregnancy complications (AOR: 1.08, 95% CI: 1.05–1.11) were more likely to have LBW children. Maternal food diversity was found to a protective factor against children’s LBW occurrence. Compared to mothers who had low diversity in their meals, mothers who consumed medium (AOR: 0.94, 95% CI: 0.91–0.98) or high (AOR: 0.94, 95% CI: 0.89–0.99) diversity food were less likely to experience LBW babies. The present study also revealed that the LBW of children was significantly determined by nutritional status (measured from BMI) and anemia levels of mothers. Compared to women with normal BMI, underweight women were more likely (AOR: 1.28, 95% CI: 1.23–1.32) and overweight/obese women were less likely (AOR: 0.92, 95% CI: 0.88–0.97) to have LBW children. Likewise, anemic women were associated with an increased likelihood of LBW children (AOR: 1.06, 95% CI: 1.03–1.09). Maternity care services were also found to be influential factors in reducing the risk of LBW children. Women who attended ≥4 ANC visits (AOR: 0.84, 95% CI: 0.80–0.88), took iron tablets/syrup during pregnancy (AOR: 0.94, 95% CI: 0.90–0.98), and delivered in a public health facility (AOR: 0.84, 95% CI: 0.79–0.88) were less likely to have LBW babies. Distance to the health facility also had a significant impact on LBW of children. Women who reported big problems regarding distance to health facility were 10% increased likelihood of having LBW children (AOR: 1.10, 95% CI: 1.06–1.14) as compared to those who reported no such problems.

**Table 2:** Maternal factors associated with low birth weight of children in India, NFHS-4.

Factors	Model 1			Model 2			Model 3		
	OR	95% CI	*p*-value	OR	95% CI	*p*-value	Adjusted OR	95% CI	*p*-value
**Mother-related factors**									
Pregnancy termination									
Miscarriage	1.05	1.00–1.11	0.056	1.07	1.01–1.13	0.018	1.05	0.99–1.11	0.076
Abortion	1.06	0.97–1.15	0.199	1.07	0.99–1.17	0.091	1.08	0.99–1.18	0.068
Stillbirth	1.26	1.10–1.44	0.001	1.27	1.11–1.46	0.001	1.20	1.04–1.38	0.012
None (Ref.)									
Any sign of pregnancy complications									
No (Ref.)									
Yes	1.08	1.05–1.11	<0.001	1.08	1.05–1.11	<0.001	1.08	1.05–1.11	<0.001
Maternal food diversity index									
Low (Ref.)									
Medium	0.90	0.86–0.93	<0.001	0.92	0.89–0.96	<0.001	0.94	0.91–0.98	0.004
High	0.88	0.84–0.93	<0.001	0.92	0.87–0.96	0.001	0.94	0.89–0.99	0.015
Maternal BMI									
Underweight	1.34	1.29–1.38	<0.001	1.33	1.28–1.37	<0.001	1.28	1.23–1.32	<0.001
Normal (Ref.)									
Overweight/obese	0.85	0.82–0.89	<0.001	0.87	0.83–0.90	<0.001	0.92	0.88–0.97	0.001
Maternal anemia									
Not anemic (Ref.)									
Anemic	1.06	1.03–1.09	<0.001	1.06	1.03–1.09	<0.001	1.06	1.03–1.09	<0.001
**Health care factors**									
Number of ANC visits									
None (Ref.)									
1–3				0.87	0.83–0.91	<0.001	0.87	0.83–0.91	<0.001
≥4				0.82	0.78–0.86	<0.001	0.84	0.80–0.88	<0.001
Uptake of iron tablets/syrup during pregnancy									
No (Ref.)									
Yes				0.92	0.88–0.95	<0.001	0.94	0.90–0.98	0.002
Place of delivery									
Home (Ref.)									
Public health facility				0.85	0.81–0.90	<0.001	0.84	0.79–0.88	<0.001
Private health facility				0.92	0.87–0.97	0.004	0.97	0.91–1.03	0.314
Distance to health facility									
No problem (Ref.)									
Big problem				1.16	1.12–1.20	<0.001	1.10	1.06–1.14	<0.001
Not a big problem				1.09	1.05–1.12	<0.001	1.04	1.01–1.08	0.017
**Covariates**									
Women's age (years)									
15–24 (Ref.)									
25–34							0.92	0.89–0.96	<0.001
35–49							0.96	0.90–1.02	0.215
Age at marriage (years)									
<18							1.02	0.98–1.05	0.326
≥18 (Ref.)									
Place of residence									
Urban (Ref.)									
Rural							0.88	0.85–0.91	<0.001
Caste									
Forward caste (Ref.)									
Scheduled caste							1.05	1.00–1.11	0.034
Scheduled tribe							0.93	0.88–0.99	0.013
Other backward classes							0.96	0.93–1.01	0.087
Religion									
Hindu (Ref.)									
Muslim							0.95	0.90–0.99	0.026
Others							0.79	0.74–0.84	<0.001
Birth order									
1 (Ref.)									
2							0.86	0.83–0.89	<0.001
≥3							0.86	0.82–0.90	<0.001
Maternal education									
No education (Ref.)									
Primary							1.02	0.98–1.08	0.337
Secondary							0.91	0.88–0.95	<0.001
Higher							0.73	0.68–0.77	<0.001
Wealth quintile									
Poorest (Ref.)									
Poorer							0.97	0.93–1.02	0.256
Middle							0.93	0.89–0.98	0.008
Richer							0.92	0.87–0.98	0.008
Richest							0.77	0.72–0.82	<0.001
Region									
North (Ref.)									
Central	1.00	0.96–1.04	0.892	0.97	0.93–1.01	0.151	0.90	0.86–0.94	<0.001
East	0.76	0.73–0.79	<0.001	0.72	0.69–0.76	<0.001	0.65	0.62–0.69	<0.001
Northeast	0.50	0.47–0.53	<0.001	0.50	0.47–0.52	<0.001	0.49	0.46–0.53	<0.001
West	0.97	0.92–1.02	0.215	0.96	0.90–1.01	0.115	0.88	0.83–0.94	<0.001
South	0.84	0.80–0.89	<0.001	0.86	0.82–0.91	<0.001	0.81	0.76–0.85	<0.001

Notes: Ref.: Reference category; OR: Odds ratio; CI: Confidence interval.

Regarding demographic factors, women ages 25–34 (AOR: 0.92, 95% CI: 0.89–0.96) were less likely to have LBW babies as compared to women ages 15–24 years. Participants living in rural areas were less likely to have LBW babies (AOR: 0.88, 95% CI: 0.85–0.91) as compared to their urban counterparts. Concerning caste and religion groups, scheduled tribe (AOR: 0.93, 95% CI: 0.88–0.96) and Muslim (AOR: 0.95, 95% CI: 0.90–0.99) children had a lower risk of LBW in the adjusted analysis. The probability of LBW children reduced with higher-order birth. Maternal education was found to be a protective factor against children’s LBW occurrence. Compared to women who had no education, women with secondary (AOR: 0.91, 95% CI: 0.88–0.95) and higher education (AOR: 0.73, 95% CI: 0.68–0.77) were less likely to have LBW babies. Women from the richest quintile had reduced odds of LBW babies (AOR: 0.77, 95% CI: 0.72–0.82) as compared to women from the poorest quintile. The geographical region also had a significant influence on the incidence of LBW babies. The results revealed that children resided in the central (AOR: 0.90, 95% CI: 0.86–0.94), east (AOR: 0.65, 95% CI: 0.62–0.69), northeast (AOR: 0.49, 95% CI: 0.46–0.53), west (AOR: 0.88, 95% CI: 0.83–0.94), and south (AOR: 0.81, 95% CI: 0.76–0.85) regions were less likely to experience LBW as compared to children from the northern region of the country.

## Discussion

India has initiated several reproductive healthcare programs for the improvement of maternal and newborn health status. Despite these efforts, a high proportion of children are born with LBW, leading to an alarming rate of child mortality in the country. We used a sub-sample of the NFHS-4 data where about 17.5% of children are found to be born with LBW, which is similar to the reported prevalence of LBW (18%) that was estimated from all living children under the age of five years in India [6]. However, the occurrence of LBW among all living under-five children was reduced by only 4% during the past decade (from 22% in 2005–06 to 18% in 2015–16) [14]. This study has identified several key maternal factors and other covariates that were significantly associated with LBW of children.

The findings of this study indicate that maternal factors are significantly associated with LBW of children. Concerning reproductive indicators, women’s prior history of stillbirth and complications during pregnancy increased the likelihood of having LBW children by 20% and 8%, respectively. Adverse reproductive health of mothers may result in restricted fetus growth. Evidence has found that the risk of LBW deepens when pregnancy occurs within 6 months of previous birth [15]. A possible explanation lies in the role of contraceptive use where the inter-pregnancy interval is generally lower among those women who are not using contraceptives that can eventually lead to a greater risk of LBW babies. Additionally, contraceptive use may increase knowledge and awareness about reproductive health care through interaction with professional health workers and that could also have a positive influence on maternal healthcare-seeking. A recently conducted study in India revealed that women who use contraceptives are more likely to attend ANC services [16]. In an agreement with previous studies [13,17], the present study affirmed that adequate utilization of ANC was associated with a decreased risk of LBW babies. Routine examination of the mother and fetus and practicing good health habits during pregnancy may help to reduce adverse pregnancy outcomes including LBW. Moreover, uptake of iron tablets or syrup during pregnancy was also associated with decreased odds of LBW babies. An unmatched cross-control study in Ghana reported that women who never took iron supplementation during pregnancy were associated with threefold increased odds of having LBW babies [18]. We found that the place of delivery had a significant association with LBW in which women who delivered in a public health institution were less likely to have LBW children. It is assumed that women who delivered in a health institution are more likely to visit health centre for their health check-ups and other healthcare services during the pregnancy period that would have a positive impact on fetus growth. A study carried out in Bangladesh found twofold increased odds of having LBW babies among those women who delivered a baby in the home [12]. The likelihood of LBW was more common among mothers who reported that the distance to the health facility was a big problem. Difficulties in accessing healthcare regarding distance to the health facility are acted as a strong barrier to adequate health care utilization among pregnant mothers. An earlier study conducted in India using NFHS-4 data indicated that women’s reported problems in visiting health facilities were associated with underutilization of ANC services, which further may result in LBW of infants [16].

A noteworthy finding in this study is that the food diversity of women had a significant impact on the birth weight of infants. The likelihood of LBW decreased with an increasing level of food diversity. This result is in accordance with the findings from Ghana where mothers who consumed diversified and nutritious foods were less likely to have LBW babies [19]. Similarly, an Indian study reported that the intake of high protein foods by mothers (i.e., consumption of dairy products) significantly reduces the risk of LBW [10]. Proper nutritious foods are essential for a healthy pregnancy as a healthy diet during pregnancy stimulates the baby’s growth and development. Deficiencies in protein, energy, and micronutrients result in depletion of body mass that can further lead to LBW of infants. Furthermore, our study revealed that underweight women were at a greater risk of LBW babies. This finding is corroborated with many earlier studies conducted in India and elsewhere [8,10,11]. Nevertheless, the magnitude of risk may vary depending on the healthcare management and monitoring system in different geographical settings [8]. A recently conducted study in India found that stunted mothers had a 30% higher likelihood of LBW babies [10]. Similarly, Sharma et al. [20] also found that mothers’ height below 146 cm increased the risk of LBW babies by 65%. It is evidenced that the poor nutrition of mothers negatively associated with fetal growth. In line with an earlier study [21], the present study also indicates that anemic women were associated with an elevated risk of LBW babies. Poor nutritional status of the mother leads to depletion of nutrient reserves that may further affect fetal growth. It is important to note that the birth weight of infants is determined by pre-pregnancy and during pregnancy nutritional status. However, the current study investigated the association of nutritional status and anemia of mothers with children’s LBW using women’s biomarkers’ information collected at the time of the survey. Further research is warranted using longitudinal data to establish a more robust relationship between mothers’ nutritional status and LBW of children.

Several demographic (e.g., women’s age and age at marriage) and socio-economic characteristics (e.g., place of residence, caste, religion, birth order, education, wealth status, and geographical region) were significantly associated with LBW of children. Among socio-demographic factors, maternal education and household wealth status were found to be protective factors against LBW of children. The odds of having LBW decreased with an increasing level of maternal education. This finding is in line with several previous studies conducted in India and other developing countries [10,12,17,22,23]. Educated women generally have greater access to healthcare facilities and more informed about the risk of inadequate healthcare utilization compared to the uneducated ones. Similarly, the probability of LBW babies decreased from bottom to upper quintiles of household wealth. Economically well-off families would go better healthcare facilities and more informed about the risk of inadequate healthcare-seeking because richer women are more likely to be educated. Moreover, richer families can afford a proper and nutritious diet during pregnancy period. Therefore, the chances of occurring LBW babies become lower among richer women.

The findings of this study should be considered in light of some limitations. The information regarding the birth weight of children was collected either from a written record or mother’s recall. Therefore, birth weight information from the mother’s recall is prone to recall bias and social desirability. Nonetheless, to minimize the recall bias in the study, we restricted our sample for the most recent birth of women in the past five years preceding the survey. Moreover, there is a large number of missing cases in birth weight data because many children were not weighed at birth, or respondents were refused to answer. We could not assess the causal relationship between the outcome variable and predictors due to the cross-sectional nature of the study design. This study could not assess potential factors mediating the association between maternal characteristics and LBW in children. Future research is needed using longitudinal data to understand the mechanism behind the high occurrence of LBW children in India. Despite these limitations, this study provides important insights on maternal factors associated with LBW in children, which may be useful for policymaking to reduce the incidence of LBW in Indian context.

## Conclusion

The findings of this study indicate that several maternal factors are significantly associated with the occurrence of LBW babies. Interventions are needed for adequate ANC utilization, improvement in public facility-based delivery, iron supplementation to reduce anemia, and the intake of balanced energy-protein diet among pregnant mothers. Policymakers and stakeholders are also suggested to design effective policies and programs to address the poor reproductive health of women that would further help to reduce the risk of LBW babies. Additional efforts should be taken among socio-economically vulnerable groups of women to address adverse pregnancy and birth outcomes including LBW.

## Supporting information

S1 Table Sample distribution of food items, NFHS-4. (DOCX)

S2 Table Correlation matrix of food items. (DOCX)

S3 Table Multicollinearity test for explanatory variables. (DOCX)
