# Correction: Maternal determinants of low birth weight among Indian children: Evidence from the National Family Health Survey-4, 2015-16

**DOI:** 10.1371/journal.pone.0250140

**Published:** 2021-04-08

**Authors:** Ankita Zaveri, Pintu Paul, Jay Saha, Bikash Barman, Pradip Chouhan

The order of Figs 1 and 2 is switched.

**Fig 1:**
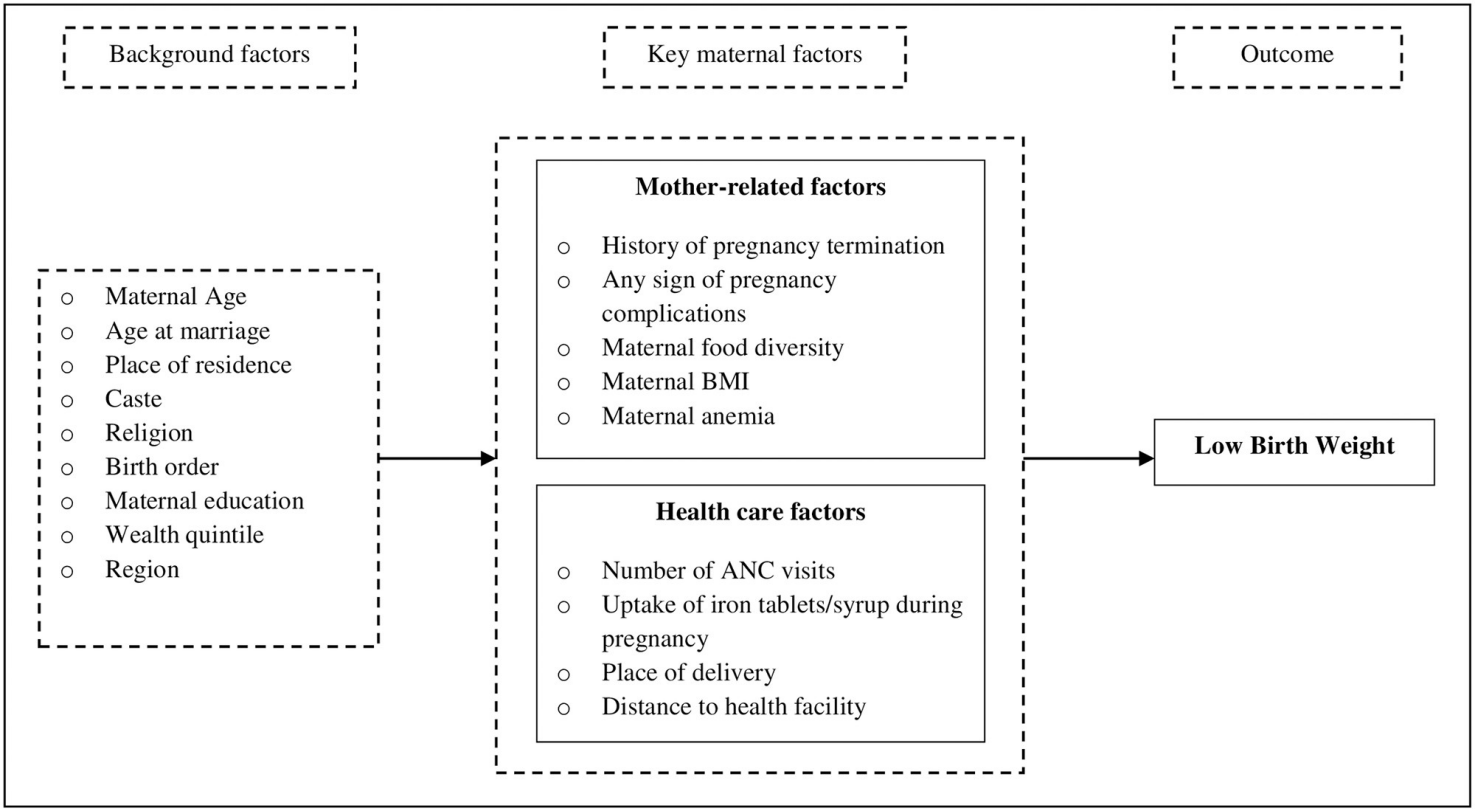
Conceptual framework showing maternal determinants of low birth weight.

**Fig 2:**
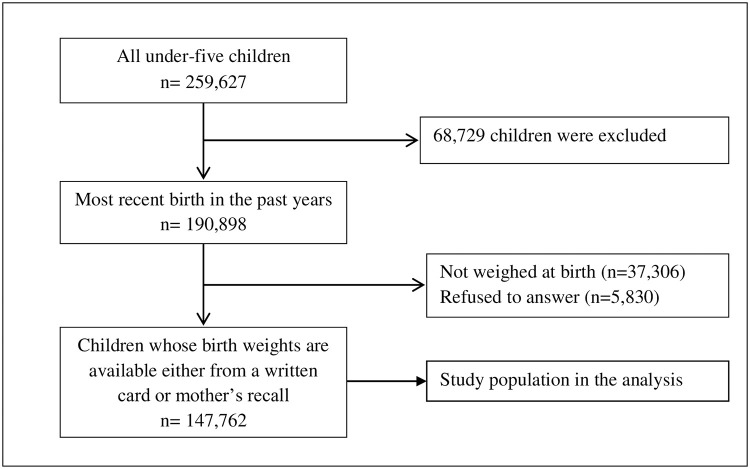
Selection of study participants, NFHS-4.
