# Supplementary material for: Maternal determinants of low birth weight among Indian children: Evidence from the National Family Health Survey-4, 2015-16
Source: PLoS One. 2020 Dec 31;15(12):e0244562. doi: 10.1371/journal.pone.0244562 (PMC7774977; doi:10.1371/journal.pone.0244562)
Supplement: S1 Table — (DOCX) [file pone.0244562.s001.docx]

**S1 Table**: Sample distribution of food items, NFHS-4

| Food items | Frequency | Percent |
| --- | --- | --- |
| **Milk** |  |  |
| Never | 16,488 | 7.1 |
| Daily | 72,615 | 42.7 |
| Weekly | 44,714 | 23.5 |
| Occasionally | 57,081 | 26.8 |
| **Pulses** |  |  |
| Never | 869 | 0.5 |
| Daily | 81,230 | 45.2 |
| Weekly | 85,627 | 44.8 |
| Occasionally | 23,172 | 9.5 |
| **Green vegetable** |  |  |
| Never | 499 | 0.3 |
| Daily | 92,771 | 47.3 |
| Weekly | 69,461 | 38.2 |
| Occasionally | 28,167 | 14.3 |
| **Fruits** |  |  |
| Never | 5,027 | 2.8 |
| Daily | 18,900 | 11.3 |
| Weekly | 58,327 | 32.0 |
| Occasionally | 108,644 | 53.9 |
| **Egg** |  |  |
| Never | 51,729 | 26.8 |
| Daily | 6,398 | 4.2 |
| Weekly | 64,503 | 37.4 |
| Occasionally | 68,268 | 31.7 |
| **Fish** |  |  |
| Never | 62,387 | 32.3 |
| Daily | 7,391 | 5.1 |
| Weekly | 50,775 | 28.9 |
| Occasionally | 70,345 | 33.7 |
| **Chicken** |  |  |
| Never | 55,914 | 29.0 |
| Daily | 2,408 | 1.1 |
| Weekly | 55,337 | 31.6 |
| Occasionally | 77,239 | 38.3 |
| **Fried food** |  |  |
| Never | 8,175 | 4.4 |
| Daily | 21,651 | 9.3 |
| Weekly | 63,789 | 35.7 |
| Occasionally | 97,283 | 50.7 |
| **Aerated drinks** |  |  |
| Never | 34,066 | 17.5 |
| Daily | 8,266 | 4.1 |
| Weekly | 32,453 | 18.0 |
| Occasionally | 116,113 | 60.4 |
