# Supplementary material for: Maternal determinants of low birth weight among Indian children: Evidence from the National Family Health Survey-4, 2015-16
Source: PLoS One. 2020 Dec 31;15(12):e0244562. doi: 10.1371/journal.pone.0244562 (PMC7774977; doi:10.1371/journal.pone.0244562)
Supplement: S2 Table — (DOCX) [file pone.0244562.s002.docx]

**S2 Table**: Correlation matrix of food items

| Food items | Pulses | Milk | Fruits | Vegetable | Egg | Fish | Chicken | Fried food | Aerated drinks |
| --- | --- | --- | --- | --- | --- | --- | --- | --- | --- |
| Pulses | 1 |  |  |  |  |  |  |  |  |
| Milk | 0.1906 | 1 |  |  |  |  |  |  |  |
| Fruits | 0.1527 | 0.2901 | 1 |  |  |  |  |  |  |
| Vegetable | 0.2426 | 0.0780 | 0.1517 | 1 |  |  |  |  |  |
| Egg | 0.0964 | 0.0428 | 0.2254 | 0.1720 | 1 |  |  |  |  |
| Fish | 0.0779 | -0.0081 | 0.1768 | 0.1456 | 0.5868 | 1 |  |  |  |
| Chicken | 0.0446 | 0.0389 | 0.1954 | 0.1255 | 0.5700 | 0.5893 | 1 |  |  |
| Fried food | 0.0998 | 0.0457 | 0.1913 | 0.1417 | 0.2655 | 0.2898 | 0.2399 | 1 |  |
| Aerated drinks | 0.0830 | 0.1782 | 0.2561 | 0.0617 | 0.1159 | 0.0964 | 0.1306 | 0.2571 | 1 |
