# Supplementary material for: Maternal determinants of low birth weight among Indian children: Evidence from the National Family Health Survey-4, 2015-16
Source: PLoS One. 2020 Dec 31;15(12):e0244562. doi: 10.1371/journal.pone.0244562 (PMC7774977; doi:10.1371/journal.pone.0244562)
Supplement: S3 Table — (DOCX) [file pone.0244562.s003.docx]

**S3 Table**: Multicollinearity test for explanatory variables

| Factors | VIF | 1/VIF |
| --- | --- | --- |
| **Mother-related factors** |  |  |
| Pregnancy termination | 1.01 | 0.985862 |
| Miscarriage | 1.01 | 0.990057 |
| Abortion | 1.00 | 0.995237 |
| Stillbirth |  |  |
| None (Ref.) |  |  |
| Any signs of pregnancy complications |  |  |
| No (Ref.) |  |  |
| Yes | 1.02 | 0.984005 |
| Maternal food diversity index |  |  |
| Low (Ref.) |  |  |
| Medium | 1.79 | 0.558012 |
| High | 1.95 | 0.513315 |
| Maternal BMI |  |  |
| Underweight | 1.11 | 0.903531 |
| Normal (Ref.) |  |  |
| Overweight | 1.16 | 0.864340 |
| Maternal anemia |  |  |
| Not anemic (Ref.) |  |  |
| Anemic | 1.03 | 0.971495 |
| **Health care factors** |  |  |
| Number of ANC visits |  |  |
| None (Ref.) |  |  |
| 1–3 | 2.80 | 0.357709 |
| ≥4 | 3.10 | 0.322128 |
| Uptake of iron tablets/syrup during pregnancy |  |  |
| No (Ref.) |  |  |
| Yes | 1.09 | 0.913534 |
| Place of delivery |  |  |
| Home (Ref.) |  |  |
| Public health facility | 3.50 | 0.285707 |
| Private health facility | 3.88 | 0.257686 |
| Distance to health facility |  |  |
| No problem (Ref.) |  |  |
| Big problem | 1.49 | 0.669157 |
| Not a big problem | 1.38 | 0.723823 |
| **Covariates** |  |  |
| Women's age (years) |  |  |
| 15–24 (Ref.) |  |  |
| 25–34 | 1.58 | 0.631848 |
| 35–49 | 1.55 | 0.644242 |
| Age at marriage (years) |  |  |
| <18 | 1.28 | 0.782620 |
| ≥18 (Ref.) |  |  |
| Place of residence |  |  |
| Urban (Ref.) |  |  |
| Rural | 1.35 | 0.743036 |
| Caste |  |  |
| Forward caste (Ref.) |  |  |
| Scheduled caste | 1.78 | 0.561708 |
| Scheduled tribe | 2.06 | 0.485659 |
| Other backward classes | 1.94 | 0.515744 |
| Religion |  |  |
| Hindu (Ref.) |  |  |
| Muslim | 1.12 | 0.889944 |
| Others | 1.52 | 0.655819 |
| Birth order |  |  |
| 1 (Ref.) |  |  |
| 2 | 1.45 | 0.691479 |
| ≥3 | 2.04 | 0.489075 |
| Maternal education |  |  |
| No education (Ref.) |  |  |
| Primary | 1.47 | 0.682297 |
| Secondary | 2.29 | 0.437621 |
| Higher | 2.19 | 0.456913 |
| Wealth quintiles |  |  |
| Poorest (Ref.) |  |  |
| Poorer | 1.87 | 0.535985 |
| Middle | 2.21 | 0.452393 |
| Richer | 2.64 | 0.379262 |
| Richest | 3.43 | 0.291738 |
| Region |  |  |
| North (Ref.) |  |  |
| Central | 1.94 | 0.51520 |
| East | 2.00 | 0.498924 |
| Northeast | 2.01 | 0.498228 |
| West | 1.44 | 0.694017 |
| South | 1.72 | 0.581754 |

Ref.: Reference category
